# Nurturing care indicators for the Brazilian Early Childhood Friendly Municipal Index (IMAPI)

**DOI:** 10.1111/mcn.13155

**Published:** 2021-05-04

**Authors:** Gabriela Buccini, Jéssica Pedroso, Stefanie Coelho, Gabriel Ferreira de Castro, Juracy Bertoldo, Alberto Sironi, Joao Gondim, Sonia Isoyama Venancio, Rafael Pérez‐Escamilla, Marcos Ennes Barreto, Muriel Bauermann Gubert

**Affiliations:** ^1^ Department of Environmental Health University of Nevada, Las Vegas 4700 S. Maryland Parkway, Suite #335, Mail Stop #3063 Las Vegas Nevada 89119 USA; ^2^ Department of Nutrition University of Brasília Brasilia Brazil; ^3^ Federal University of Bahia Salvador Brazil; ^4^ Instituto de Saúde Secretaria de Estado da Saúde de São Paulo São Paulo Brazil; ^5^ Department of Social and Behavioral Sciences, Yale School of Public Health Yale University New Haven Connecticut USA

**Keywords:** Brazil, child development, cities, index, indicator, monitoring, nurturing care

## Abstract

The Nurturing Care Framework (NCF) calls for establishing a global monitoring and accountability systems for early childhood development (ECD). Major gaps to build low‐cost and large‐scale ECD monitoring systems at the local level remain. In this manuscript, we describe the process of selecting nurturing care indicators at the municipal level from existing routine information systems to develop the Brazilian Early Childhood Friendly Index (IMAPI). Three methodological steps developed through a participatory decision‐making process were followed. First, a literature review identified potential indicators to translate the NCF domains. Four technical panels composed of stakeholders from federal, state and municipal levels were consulted to identify data sources, their availability at the municipal level and the strengths and weakness of each potential indicator. Second, national and international ECD experts participated in two surveys to score, following a SMART approach, the expected performance of each nurturing care indicator. This information was used to develop analytical weights for each indicator. Third, informed by strengths and weaknesses pointed out in the previous steps, the IMAPI team reached consensus on 31 nurturing care indicators across the five NCF domains (Good health [*n* = 14], Adequate nutrition [4], Responsive caregiving [1], Opportunities for early learning [7] and Security and safety [4]). IMAPI represents the first attempt to select nurturing care indicators at the municipal level using data from existing routine information systems.

**Key messages**

- The Nurturing Care Framework (NCF) calls for establishing monitoring systems for early childhood development (ECD) to achieve the 2030 Sustainable Development Goals.
- A three‐step participatory methodological process was designed and piloted to select municipal‐level nurturing care indicators using a routine information system.
- Brazil is an important country to identify municipal‐level nurturing care indicators because the implementation of ECD programmes happens across its 5570 municipalities in the context of great inequities.
- The Brazilian Early Childhood Friendly Municipal Index (IMAPI) represents the first attempt to select nurturing care indicators at the municipal level using existing routine information systems.

## 1 INTRODUCTION

Optimal early childhood development (ECD) follows a gradual process that is contingent on children and their families having access to nurturing care environments needed by children to reach their full motor, language, socio‐emotional and cognitive development (Black et al., 2017; Shonkoff et al., 2012). Nurturing care is defined as an environment that is responsive, emotionally supportive, sensitive to children's health and nutritional needs and developmentally appropriate and stimulating, with opportunities for play and exploration, protecting children from adversities (Britto et al., 2017). The WHO/UNICEF/World Bank Nurturing Care Framework (NCF) translated this evidence into five essential domains (i.e. Good health, Adequate nutrition, , Responsive caregiving, Opportunities for early learning, and Security and safety) and calls for establishing global monitoring based on the principles of equity, integrity and social justice to achieve the 2030 Sustainable Development Goals (Black et al., 2017; Morris et al., 2017; Richter et al., 2016).

Efforts to build large‐scale ECD monitoring systems based on the NCF have happened to a limited extent at the national level (World Health Organization, World Bank Group, & United Nations Children's Fund, 2019), and very few countries have done it at the state levels. At the national level, the most visible effort at mapping national data comes from an effort launched in 2019 by the United Nations Children's Fund (UNICEF) in partnership with Countdown to 2030 for Women's, Children's and Adolescents' Health. It involves mapping ‘common indicators’ into country profiles following the NCF. Currently, 138 low‐ and middle‐income countries have NCF country profiles. Although undoubtedly this initiative is key for global ECD advocacy, a major gap remains on how to monitor nurturing care indicators at the state and municipal levels, or even smaller geopolitical units in the countries (Köhler & Eriksson, 2018). Considering the challenge for countries to collect primary data to monitor the complex and multisectoral NCF, the use of public domain routine information systems from different sectors including education, health, nutrition, social security, law enforcement and child protection presents a great opportunity to build a useful and sustainable large‐scale ECD monitoring system. However, this is challenging because monitoring NCF requires integrating large amounts of reliable data at the municipal level (Cavallera et al., 2019; World Health Organization, United Nations Children's Fund, World Bank Group, Early Childhood Development Action Network, & Partnership for Maternal Newborn and Child Health, 2019).

In this context, Brazil, which is the largest and the most populous country in Latin America (with 211 million inhabitants), provides an important opportunity for testing a municipal‐level ECD index because the operationalization of ECD programmes occurs across 5570 municipalities in the context of great socio‐economic and environmental disparities (Aristides dos Santos et al., 2019). Hence, translating and operationalizing the NCF into integrated ECD strategies through effective actions according to the needs and specific determinants of each community is a major challenge. Furthermore, over the past 33 years, three legal frameworks aimed at guaranteeing the rights of children have been created—the Federal Constitution of 1988, the 1990 Child and Adolescent Statute and the 2016 Legal Framework for Early Childhood. As a result, in recent years, the ECD agenda in the country has expanded greatly, and investments made in programmes focused on ECD, such as Criança Feliz (‘Happy Child’), a home visiting programme that has already reached about 3000 Brazilian municipalities (Buccini et al., 2021; Girade, 2018). Furthermore, Brazil has a large volume of high‐quality public information databases on health, education and social development (including cash transfer programmes); however, data integration for nurturing care monitoring purposes does not exist at any level of government. Thus, we aimed to describe the process of identifying and selecting nurturing care indicators at the municipal level from existing Brazilian databases to develop the Brazilian Early Childhood Friendly Municipal Index (IMAPI, ‘Índice Município Amigo da Primeira Infância*’*).

## 2 METHODS

**Figure 1:**
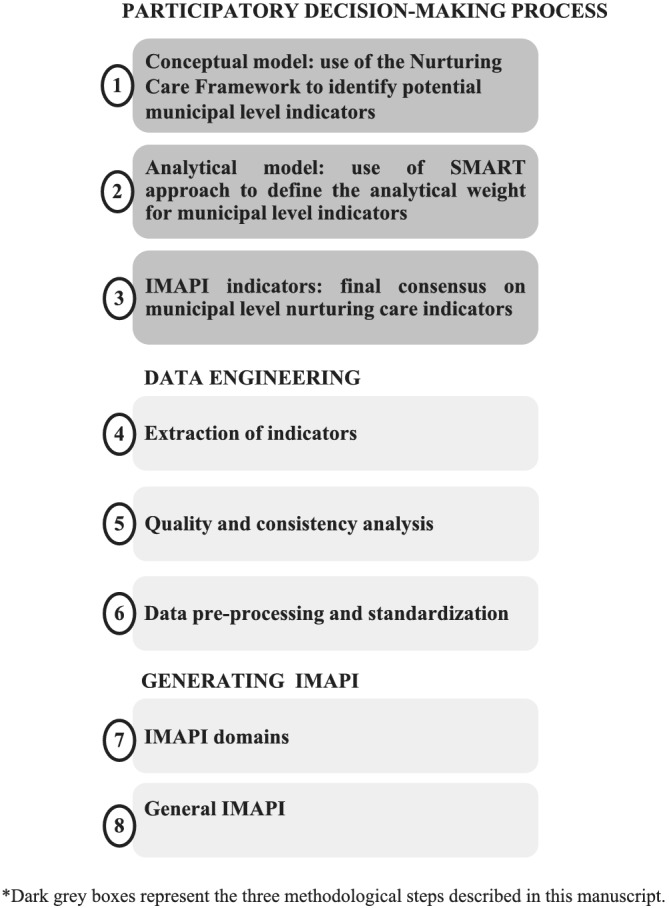
Systematic methodology to develop the Brazilian Early Childhood Friendly Municipality Index (IMAPI)

IMAPI was developed following a systematic methodology (Figure 1). In this manuscript, we describe the participatory decision‐making process used in the first three methodological steps to identify and select nurturing care indicators at the municipal level. The name of indicators is reported as ‘indicator name’ throughout.

### 2.1 Participatory decision‐making process

**Figure 2:**
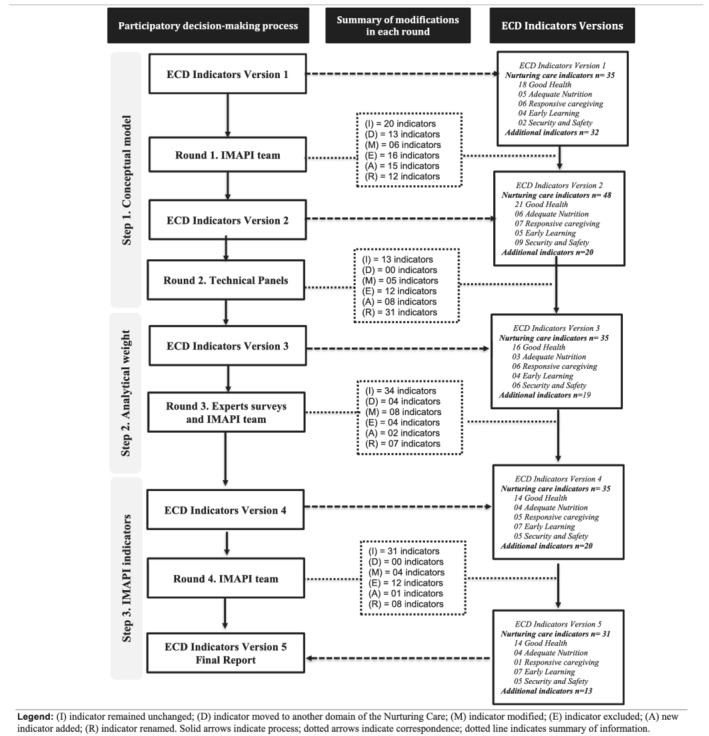
Participatory decision‐making process used in the three methodological steps to identify and reach consensus on nurturing care indicators at the municipal level in Brazil

A multisectoral and participatory decision‐making process was designed to facilitate group communication and to reach consensus on the nurturing care indicators in Brazil. Consultative participatory methods have been used to enhance transparency, accountability, equity and efficiency in the decision‐making process, which are adequate to identify and select nurturing care indicators (Devente et al., 2016; Elwyn et al., 2017; Okoli & Pawlowski, 2004). In this study, the participatory decision‐making process included consultations with participants within each of the three methodological steps (Figure 2).

#### 2.1.1 Participants selection and characteristics

Technical stakeholders, experts and the IMAPI team engaged in the different steps of the participatory decision‐making process (Figure 2). Detail on participants' institutions is described in Table S1. In Step 1, technical panels were convened to identify nurturing care indicators as well as routine information systems available at the municipal level. A formal invitation describing the technical panel's goals and characteristics of individuals who could serve as a participant was sent to government institutions (*n* = 15) as well as non‐government institutions (*n* = 4). Each institution could determine and recommend one or more participants based on the technical panels' goals. Although the number of participants varied across technical panels, all invited institutions sent at least one participant. Technical panel participants were individuals that managed or had in‐depth knowledge of national databases with information about health, education and social development in Brazil. In Step 2, expert surveys were designed to validate the selection of municipal‐level nurturing care indicators and score each of the SMART attributes to inform an evidence‐based analytical weight per indicator. Invited experts were identified within the Brazilian and international institutions and networks highly engaged with ECD programming in Brazil. Expert surveys' participants included individuals with experience in ECD and public policy, who had in‐depth knowledge of the socio‐economic, geographical and political context of Brazil. Fourteen experts were invited by email, and 12 accepted the invitation to participate. Of those, 11 experts participated in the first survey, and seven in the second survey. In Steps 1–3, the IMAPI team engaged throughout the participatory decision‐making process. Each of the four rounds started with an input statement, which consisted of a list of nurturing indicators named ‘ECD Indicators Versions 1–4’ produced by the IMAPI team. At the end of each round, this list of nurturing care indicators was revised by the IMAPI team to summarize modifications and consensus within that round, which generated an output statement, which, in turn, became the input statement for the next round. The IMAPI team was composed of the principal investigators, research assistants and senior advisors who collectively had expertise in epidemiology, maternal–child nutrition, implementation science and data science and machine learning.

##### Step 1. Conceptual model

This step aimed to use the NCF conceptual framework to identify potential nurturing care indicators at the municipal level. Initially, the first author generated an input statement with an initial list of indicators (ECD Indicators Version 1) based on a careful review of documents and technical materials measuring actions and policies for early childhood, such as Countdown 2030, World Bank, World Health Organization, NCF website and São Paulo Early Childhood Index (IPPI) (Naudeau et al., 2011; SEADE, 2017; UNICEF and & Countdown to 2030, 2019; World Health Organization, United Nations Children's Fund, & World Bank Group, 2018). At this stage, the goal was to collect the largest number of potential indicators; thus, we included indicators that contributed to any of one of the five domains of the NCF, operationalized as follows: *Good health* (indicators related to healthcare from prenatal to the first years of life), *Adequate nutrition* (indicators related to the promotion of healthy eating, access to food and child nutrition), *Responsive caregiving* (indicators related to family skills and child care in the home environment), *Opportunities for early learning* (indicators related to the access and quality of formal education) and *Security and safety* (indicators related to protections or vulnerabilities to which the child may be exposed in the family or community environment). Indicators considered important for placing the NCF into the diverse municipal contexts were allocated to the category of ‘additional indicators’ (e.g. population size and the region of the country), which also underwent revisions, additions or removal following the same methods followed for the selection of the nurturing care indicators. Second, *in Round 1*, the IMAPI team discussed the ‘ECD Indicators Version 1’ based on the following questions: (i) Does the indicator evaluate the enabling environment for ECD in one of the five domains of NCF? (ii) Does the indicator complement information about the enabling environment for ECD? (iii) Which of the five NCF domains does the indicator belong to? (iv) Does the indicator need to be modified or removed as it does not suit the Brazilian municipal context? These questions guided the IMAPI team to reach a consensus on modifications to each indicator. At the end of this round, the IMAPI team summarized the consensual modifications that generated a revised list of nurturing care indicators, the ‘ECD Indicators Version 2’ (Figure 2). Third, *Round 2* was composed of four technical panels to discuss the availability of the indicator at the municipal level, identification of data sources and calculation method (i.e. definition of the numerator and denominator to generate each indicator), as well as list possible weaknesses of each indicator and suggestions of potential new indicators. Consensus was reached when participants in the technical panels did not have more concerns or suggestions about an indicator or NCF domain. At the end of this round, the IMAPI team summarized the consensual modifications that generated the ‘ECD Indicators Version 3’ (Figure 2).

##### Step 2. Analytical weight

This step aimed to estimate an analytical weight for each municipal‐level nurturing care indicator following a SMART process. Because the NCF assumes that all domains have the same level of importance, the purpose of the analytical weighting exercise was to improve reliability when generating IMAPI indexes by giving higher performance analytical weight to indicators with better data quality attributes (Köhler, 2016; OECD, 2008). Additional indicators were not included in the calculation of IMAPI indexes; thus, they did not receive scores for analytical weight. *Round 3* was composed of two surveys for experts following a SMART approach (Centers for Disease Control, 2011). Surveys were set up into an Excel spreadsheet using the indicators listed in the ‘ECD indicators Version 3’ and were sent by email to experts. A detailed methodological note on how questions were asked and analysed can be found in Table S3. Briefly, in the **first survey**, criterion ‘S’ (Specific) assessed the specificity of each indicator allocation across the NCF domains and was confirmed by the percentage of agreement among experts. This information was used to assure appropriate weighting procedures by confirming the appropriate allocation of each indicator in the NCF domain. Experts' consensual modifications or suggestions regarding the indicators were summarized by the IMAPI team in the ‘ECD Indicators Version 4’ (Figure 2). Criterion ‘M’ (Measurable) was assessed jointly by experts and the IMAPI team. Experts ranked 1 (*lowest*) to 5 (*highest*) the importance of four quality attributes for each indicator: periodicity of data (M1 Periodicity), data source (M1 Source), access to data (M1 Access) population profile (M1 Population profile). Then, the four quality attributes were assessed for each indicator by the IMAPI team by scoring 1 (*lowest*) to 5 (*highest*) (i.e. generating weights [W] for Periodicity, Source, Access and Population profile for each indicator). Criterion M for each indicator consisted of the average of M1 and W1 of each quality attribute. The **second survey** consisted of scoring A, R and T criteria from score 0 (*lowest*) to 5 (*highest*) in relation to governance needed for modifying the nurturing care indicators for achieving greater impact on ECD outcomes at the municipal level (criterion ‘A’, Achievable), the extent to which changes in the indicators have the potential to directly impact ECD (criterion ‘R’, Relevant) and the time that improvements in an indicator would take to positively impact ECD (criterion ‘T’, Time‐bound).

**Survey analysis** informed the development of the formula to estimate the analytical weight per nurturing care indicator (OECD, 2008), as follows: *Indicator*_*weight*_ *= [criterion M + criterion A + criterion R + criterion T]/4*. Indicator analytical weight can range from 1 (*low*) to 5 (*high*) performance. The average of M, A, R and T criteria for each domain was used to identify indicators with best quality performances (i.e. greater than the mean of the domain) and worst performances (i.e. less than the mean of the domain) by NCF domains. Any indicator added after the determination of SMART analytical weights received the average analytical weight of the domain the new indicator was allocated, as indicated in Table 1.

**Table 1:** Results of the SMART methodology to generate the analytical weight for each Early Childhood Friendly Municipal Index (IMAPI) nurturing care indicator in Brazil

Nurturing Care domain	Nurturing care indicators	Criteria average				SMART analytical weight	
		M	A	R	T		
**Good health**	Prenatal care consultations	**4.66**	**4.43**	3.86	**3.86**	4.20	
	Early start of prenatal care	**4.66**	**4.29**	3.86	**3.86**	4.17	
	Congenital syphilis	4.16	**4.00**	3.86	**3.86**	3.97	
	C‐section	**4.66**	3.29	3.43	2.14	3.38	
	Home visits in the first 10 days of child's life	3.66	**4.43**	**4.43**	**4.43**	4.24
	Child hospitalization for pneumonia or gastroenteritis	3.91	3.71	3.00	3.29	3.48	
	Prematurity	**4.66**	3.71	**4.43**	**4.14**	3.99	
	Low birth weight	**4.66**	3.71	**4.43**	**4.14**	4.24	
	Child mortality	**4.66**	3.71	**4.14**	3.00	3.88	
	Preventable deaths in children under 1 year old	**4.66**	3.86	4.00	3.29	3.95	
	Adolescent pregnancy	**4.66**	3.57	**4.43**	3.29	4.24	
	Maternal mortality	**4.66**	3.57	**4.43**	2.43	3.77	
	Coverage of child immunization	3.91	**4.43**	4.00	**3.57**	3.98	
	Coverage of primary healthcare	4.16	**4.57**	**4.14**	3.00	3.97	
**Adequate nutrition**	Coverage of information on child food consumption	**3.66**	**4.14**	3.14	2.14	3.27	
	Coverage of information on child nutritional status	**3.66**	**4.00**	3.43	2.43	3.86	
	Severe household food insecurity	3.16	3.00	**4.86**	**4.43**	3.72	
	Brazilian breastfeeding and feeding strategy ^a^	**4.16**	**4.29**	3.71	2.71	3.38	
**Responsive caregiving**	Visits by national home‐visiting parenting skills programme^c^	**3.91**	**3.86**	**4.00**	**3.00**	3.69	
**Opportunities for early learning**	Coverage of daycare and preschool	4.16	**3.86**	**4.14**	**3.00**	3.79	
	Number of students per daycare professional	**4.41**	**4.00**	3.86	**3.00**	3.82	
	Number of students per preschool professional	**4.41**	**3.86**	4.00	2.71	3.75	
	Percentage of qualified daycare teachers	**4.41**	3.43	4.00	**3.00**	3.71	
	Percentage of qualified preschool teachers	**4.41**	3.29	4.00	**3.00**	3.68	
	Daycare educational resources	**4.41**	**4.14**	4.00	2.43	3.75	
	Preschool educational resources	**4.41**	**4.14**	**4.14**	2.43	3.78	
**Security and safety**	Notification of violence against children	**4.16**	**4.00**	**4.57**	**4.14**	3.94	
	Notification of violence against women	**4.16**	**4.14**	**4.43**	**3.57**	2.71	
	Coverage of the national conditional cash transfer programme^b^	**3.91**	**3.71**	**4.00**	**4.14**	4.08	
	Air pollution	3.41	3.00	2.86	1.57	4.22	
	Homicides^d^	3.91	3.71	3.97	3.36	3.74	

*Notes*: **Bold values** were equal or greater than the mean of the domain (best results), and those not in bold were less than the mean of domain (worst results). Criterion M means (good health = 4.41; adequate nutrition = 3.66; responsive caregiving = 3.91; opportunities for early learning = 4.37; security and safety = 3.91); Criterion A means (good health = 3.95; adequate nutrition = 3.86; responsive caregiving = 3.86; opportunities for early learning = 3.82; security and safety = 3.71); Criterion R means (good health = 4.03; adequate nutrition = 3.79; responsive caregiving = 4.00; opportunities for early learning = 4.02; security and safety = 3.97); Criterion T means (good health = 3.45; adequate nutrition = 2.93; responsive caregiving = 3.00; opportunities for early learning = 2.80; security and safety = 3.36).

^a^ Estratégia Amamenta e Alimenta Brasil.

^b^ Programa Bolsa Família.

^c^ Programa Criança Feliz.

^d^ indicator added after the determination of SMART analytical weights, thus it received the average analytical weight of the domain.

##### Step 3. IMAPI indicators

This step aimed to reach a final consensus on IMAPI's municipal‐level nurturing care indicators. In *Round 4*, the IMAPI team discussed the indicators listed in ‘ECD Indicators Version 4’ and summarized strengths and weaknesses for each indicator pointed out in the previous steps by the technical panels and expert surveys (Figure 2). Considering the need for at least two indicators to compute NCF domain indexes, the IMAPI team balanced the number of indicators across NCF domains, that is, if there were less than two indicators, then new potential indicators not analysed in the previous steps were considered. The IMAPI team reviewed notes on all indicators discussed in the previous steps to identify new potential indicators and reached a consensus on the best options with the senior advisors (RPE and SIV). The new potential indicators were included if they fulfilled the criteria of translating one of the NCF domain and were available at the municipal level. Based on these analyses, each indicator within each NCF domain was either included, relabelled to clarify the construct it was intended to represent or excluded due to the non‐availability of the database after multiple attempts. Importantly, data access and extraction started early in the decision‐making process, that is, since the first technical panel, allowing various attempts to request the database over the project's timeframe. During data extraction, data quality was scored by the IMAPI team through the SMART surveys following the four quality attributes for each indicator (periodicity of data, data source, type of access to data and population profile). Although data quality did not drive the decision‐making process regarding inclusion/exclusion of an indicator, it did drive how the IMAPI team defined the calculation method, that is, ways to aggregate data to improve quality. At the end of this round, the IMAPI team reached consensus on the final list of nurturing care indicators and their calculation method and generated the final report ‘ECD Indicators Version 5’ providing the following information for each indicator: (i) Name of indicator (ii) Technical definition, (iii) Theoretical justification of its influence on ECD, (iv) Calculation method, (v) Interpretation, (vi) Source of information (i.e. the database of reference) and (viii) Year of reference (i.e. the year in which the information was collected). The final set of indicators were presented to and endorsed by the senior advisors (RPE and SV).

## 3 RESULTS

Step 1. Round 1 started with 67 potential indicators identified through document review and finalized with 48 indicators allocated to NCF domains. Indicators were excluded if they did not discriminate among Brazilian municipalities. For example, in Brazil access to HIV treatment is free and universal; hence, this indicator is homogeneous across municipalities. In Round 2, after four technical panels, a total of 35 indicators were preselected across the NCF domains—Good health (*n* = 16), Adequate nutrition (*n* = 3), Responsive caregiving (*n* = 6), Opportunities for early learning (*n* = 4) and Security and safety (*n* = 6) (Figure 2).
Step 2. In Round 3, experts reclassified three indicators into another NCF domain (*criterion S, Indicator Allocation* ). Greater importance was given to the attribute population profile (i.e. population representativeness) (mean = 4.55), followed by access to data (4.45), periodicity (4.20) and data source (4.09). Most indicators had national representativeness (*n* = 24 out of 31), and if they were available for download via the Internet (*n* = 22) at least once per year (*n* = 23), they received the highest scores for population profile, data access and periodicity, respectively. Only two indicators (‘household food insecurity’ and ‘air pollution’) were cross‐sectional research projections (i.e. the use of statistical models to predict municipal‐level indicators) and for this reason received the lowest scores for data source (*criterion M, Indicator Characteristics* ). Changes in 18 indicators out of 31were considered achievable within the municipal‐level governance (criterion A, Achievable); 14 indicators were considered more relevant for changes in ECD (criterion R, Outcome Impacts); changes in 17 indicators were considered to lead to short‐term ECD outcome improvements (criterion T, Time for impact). Final analytical weights for each indicator were generated (Table 1).
Step 3. Table S2 shows in full detail the process followed to include, relabel or exclude indicators. On Round 4, examining the balance of indicators across NCF domains, the IMAPI team focused on identifying indicators for the domains with fewer indicators such as Security and safety and Responsive caregiving domains. Although we did not identify any new indicator for responsive care, we identified ‘Homicides’ as a proxy of community violence/safety, which was included in the Security and safety domain. At the end of this round, the IMAPI team reached a final consensus on 31 indicators that were allocated into the five domains of the NCF (Good health [*n* = 14], Adequate nutrition [4], Responsive caregiving [1], Opportunities for early learning [7] and Security and safety [4]) (Box 1). Thirteen additional indicators not allocated into NCF domains were also selected to characterize municipalities' context (e.g. total population and the proportion of children under 5 years old).

Box 1. Standard definitions of the selected indicators composing the Brazilian Early Childhood Friendly Municipal Index (IMAPI)

**Table:** 

Nurturing Care domain	Name of the indicator	Technical definition	Theoretical justification and influence on ECD	Calculation method	Interpretation	Source of information	Year of reference
**Good health**	Prenatal care consultations	Number of pregnant women with six or more prenatal care consultations in relation to the total number of pregnant women monitored (number of live births), by year and municipality of residence	The prenatal period is a sensitive period in the baby's formation and development. Proper prenatal care, prevention and early diagnosis of complications can affect child's development, as well as promote and protect the health of the mother and baby	(Number of women with six or more prenatal care consultations/total live births) * 100, per year and municipality of residence	An adequate number of prenatal care consultations can protect the child from suboptimal early child development	Sistema de Informação sobre Nascidos Vivos (SINASC/Ministry of Health)	2016
	Early start of prenatal care	Total pregnant women who started prenatal care in the first 12 weeks of pregnancy in relation to the total number of pregnant women monitored, by year and municipality of residence	The early start of prenatal care facilitates screening for risk factors and the possibility of treating possible complications early, promoting health and adequate fetal development	(Total pregnant women who started prenatal care at 12 weeks or less/total pregnant women monitored) * 100, per year and municipality of residence	The early start of prenatal care can protect the child from suboptimal early child development	Sistema de Informação sobre Nascidos Vivos (SINASC/Ministry of Health)	2016
	Congenital syphilis	Number of confirmed and notified cases of congenital syphilis in children under 5 years old in relation to the total number of children under 5 years old, by year and municipality of residence	Congenital syphilis can cause severe changes in the child's development, including pseudo‐paralysis of the limbs, neurological deafness and learning difficulties	(Number of confirmed and notified cases of congenital syphilis in children under 5 years old/population under 5 years old) * 10,000, per year and municipality of residence	Congenital syphilis is a risk factor for suboptimal early child development	Numerator: Sistema Nacional de Agravos de Notificação (SINAN/Ministry of Health); and denominator: Instituto Brasileiro de Geografia e estatística (IBGE)	2015
	C‐section	Number of births via C‐section in relation to the total number of births, by year and municipality of residence	High rates of elective C‐section are associated with prematurity and increased maternal and child morbimortality	(Total live births via C‐section/total live births) * 100, per year and municipality of residence	C‐section is a risk factor for suboptimal early child development	Sistema de Informação sobre Nascidos Vivos (SINASC/Ministry of Health)	2016
	Home visits in the first 10 days of child's life	Percentage of primary care teams in the municipality that participated in the second cycle of the PMAQ (national programme for improving access and quality in primary care) and reported carrying out home visits in the first 10 days of child's life	Home visits by the health team in the first 10 post‐partum days help to detect, prevent and quickly treat post‐partum and breastfeeding complications (adequate nutrition). Home visits by health teams also reflect the mother's and newborn's access and ties with the health service	(number of teams that conduct home visits in the first 10 days of child's life/total teams that participated in the PMAQ 2nd cycle) * 100, per year and municipality of residence	Home visits in the first 10 days of child's life can protect the child from suboptimal early child development	Programa Nacional de Melhoria do Acesso e da Qualidade da Atenção Básica (PMAQ 2° Ciclo)/Ministry of Health	2013/2014
	Child hospitalization for pneumonia or gastroenteritis	Percentage of children under 5 years old hospitalized for pneumonia or gastroenteritis in relation to the total number of children under 5 years old, by year and municipality of residence	Gastroenteritis and acute respiratory diseases result from a set of biological, environmental and socio‐cultural variables and indicate a high degree of vulnerability, which can cause greater risks and compromises child development. These are conditions that could be avoided or reduced by effective primary care (prevention, diagnosis and early treatment)	(Total children under 5 years old hospitalized for pneumonia or gastroenteritis/total children under 5 years old) * 100, per year and municipality of residence	Child hospitalization for pneumonia or gastroenteritis is a risk factor for suboptimal early child development	Numerator: Sistema de Informações Hospitalares (SIH/Ministry of Health) and denominator: Instituto Brasileiro de Geografia e Estatística (IBGE)	2015
	Adolescent pregnancy	Prevalence of pregnant adolescents (10–19 years), by year and municipality of residence	Adolescent pregnancy is a risky situation for the health of the adolescent and the newborn, with an increased chance of intrauterine growth retardation and developmental delays	(Number of live births to mothers aged 10–19 years old/total live births) * 100, per year and municipality of residence	Adolescent pregnancy is a risk factor for suboptimal early child development	Sistema de Informação sobre Nascidos Vivos (SINASC/Ministry of Health)	2016
	Low birth weight	Percentage of live births weighing less than 2500 g in relation to the total number of births, by year and municipality of residence	Low birth weight is a risk factor for inadequate child development, generating negative effects for mental and motor development as well as growth. It is also an indicator of poor prenatal care, maternal malnutrition, adolescent pregnancy and untreated infections	(Total live births weighing less than 2500 g/total live births) * 100, per year and municipality of residence	Low birth weight is a risk factor for suboptimal early child development	Sistema de Informação sobre Nascidos Vivos (SINASC/Ministry of Health)	2016
	Child mortality	Number of deaths of children under 5 years old for every 1000 live births, per year and municipality of residence	Most childhood deaths are concentrated in the first year of life, with a high share of perinatal causes (factors linked to pregnancy, childbirth and post‐partum), which are generally preventable. After the first year of life, mortality is associated with exposure to environmental factors, for example, lack of access to quality healthcare and other environmental stressors related to poverty. This indicator also expresses the failure in several public policies to promote healthy and complete child development	(Number of deaths of children under 5 years old/total live births) * 1,000, per year and municipality of residence	Higher child mortality rates are associated with negative exposure to environmental factors, which are risk factors for suboptimal early child development	Numerator: Sistema de Informação sobre Mortalidade (SIM/Ministry of Health) and denominator: Sistema de Informação sobre Nascidos Vivos (SINASC/Ministry of Health).	2016
	Preventable deaths in children under 1 year old	Deaths that could be prevented by the performance of healthcare services in children under 1 year old in relation to the total number of live births, by year and municipality of residence. Deaths caused by the following categories are considered ‘preventable’: (a) reducible by immunizations; (b) reducible by caring for women during pregnancy; (c) reducible by adequate care for women during childbirth; (d) reducible by actions, diagnosis and appropriate treatment; and (e) reducible by health promotion activities linked to primary healthcare	Preventable deaths occur when support services fail to identify early and correctly intervene in a given problem. Thus, a child who dies from a preventable cause is a child who has been deprived of development due to problems involving the health services of a particular municipality	(Number of preventable deaths in children under 1 year/total live births) * 1000, per year and municipality of residence	Higher numbers of preventable deaths in children under 1 year old are a reflect of failures in the support services, which are risk factors for suboptimal early child development	Numerator: Sistema de Informação sobre Mortalidade (SIM/Ministry of Health). Denominator: Sistema de Informação sobre Nascidos Vivos (SINASC/Ministry of Health)	2016
	Prematurity	Percentage of children born at less than 37 completed weeks of gestation in relation to the total number of births, by year and municipality of residence	Premature birth is a risk factor for inadequate child development in the early years as well as an indicator of poor prenatal care	(Total children born before 37 completed weeks of gestation/total live births) * 100, per year and municipality of residence	Prematurity is a risk factor for suboptimal early child development	Sistema de Informação sobre Nascidos Vivos (SINASC/Ministry of Health)	2016
	Maternal mortality	Number of women who died from causes related to pregnancy, childbirth or puerperium for every 100,000 live births, per year and municipality of residence	Maternal death can increase the risks of child mortality, morbidity and developmental delay in early childhood	(Number women who died due to causes related to pregnancy, childbirth and puerperium/number of live births) * 100,000, per year and municipality of residence	Maternal mortality is a risk factor for suboptimal early child development	Numerator: Sistema de Informação sobre Mortalidade (SIM/Ministry of Health) and denominator: Sistema de Informação sobre Nascidos Vivos (SINASC/Ministry of Health)	2016
	Coverage of child immunization	Percentage of children who received first dose of DTaP immunization (triple bacterial vaccine), by year and municipality of residence	Good vaccination coverage guarantees the maintenance of low incidences of immunopreventable illnesses that can influence children's development	(Number of doses of the first DTaP immunization (triple bacterial vaccine) /target population) * 100, per year and municipality of residence.	Child immunization is associated with low incidences of immune preventable illnesses, protecting the child from suboptimal early child development	Sistema de Informações do Programa Nacional de Imunizações (SI‐PNI/Ministry of Health)	2016
	Coverage of primary healthcare	Percentage of the population covered by primary healthcare, by year and municipality	The population coverage by primary healthcare teams reflects access to health services. Primary healthcare is responsible for several actions to prevent and promote adequate child development and early diagnosis of complications	[Number of family health teams * 3450 + (number of parameterized primary care teams + number of equivalent family health teams) * 3000] in the reference month (December) /population estimate * 100	Coverage of primary healthcare can protect the child from suboptimal early child development	Plataforma e‐Gestor Atenção Básica/Ministry of Health.	2016
**Adequate nutrition**	Coverage of information on child feeding practices	Percentage of children under 5 years old with at least one record of information on food consumption (breastfeeding, quality and diversity of the diet) in the food and nutrition surveillance system (SISVAN), by year and municipality of residence	Adequate food consumption is associated with better child growth and development. The registration of information on food consumption was used as a monitoring proxy	(Children under 5 years old with at least one record on food consumption in SISVAN/population under 5 years old) * 100, per year and municipality of residence	Coverage of information on child food consumption is a monitoring proxy of an adequate food consumption, which can protect the child from suboptimal early child development	Numerator: Sistema de Vigilância Alimentar e Nutricional (SISVAN/Ministry of Health) and denominator: Instituto Brasileiro de Geografia e Estatística (IBGE)	2015
	Severe household food insecurity	Estimated prevalence of severe food insecurity in a given municipality. Severe food insecurity is characterized by children's quantitative reduction in food, disruption in eating patterns resulting from lack of food and hunger caused by the inability to buy food due to the lack of money	Children exposed to hunger in the home environment are very likely to experience developmental delays. The prediction of severe food insecurity was used as a proxy for hunger in the municipality	Estimated prevalence of severe food insecurity in the municipality	Severe household food insecurity is a risk factor for suboptimal early child development	Gubert MB, Pérez‐Escamilla R. Severe food insecurity in Brazilian Municipalities, 2013. DOI: 10.1590/1413‐812320182310.265120161	2013
	Brazilian breastfeeding and feeding strategy^a^	The indicator Brazilian breastfeeding and feeding strategy (EAAB) is an indicator composed a workshop and/or training for instructors and/or certification of the basic health unit in the municipality	The practice of breastfeeding and the introduction of healthy complementary feeding are associated with adequate child development. The Brazilian breastfeeding and feeding strategy aims to qualify the actions to promote breastfeeding and healthy complementary food, improving the skills and abilities of health professionals for these activities, which should be monitored	[(∑ workshop held, training instructors, certified health unit in the municipality)/3]	Brazilian breastfeeding and feeding strategy qualifies the actions to promote breastfeeding and healthy complementary food, which can protect the child from suboptimal early child development	Coordenação‐Geral de Alimentação e Nutrição (CGAN)/Departamento de Atenção Básica (DAB)/Ministry of Health	*2013–2019
	Coverage of information on child nutritional status	Percentage of children under 5 years of age with at least one record of information on nutritional status (BMI/age) in the food and nutrition surveillance system (SISVAN), by year and municipality of residence	Adequate nutritional status is associated with better child development and, thus, must be monitored. The coverage of recorded information about nutritional status was used as a monitoring proxy	(Children under 5 years old with at least one record of nutritional status in SISVAN/population under 5 years old) * 100, per year and municipality of residence	Coverage of information on child nutritional status is a monitoring proxy of an adequate nutritional status, which can protect the child from suboptimal early child development	Numerator: Sistema de Vigilância Alimentar e Nutricional (SISVAN/Ministry of Health) and denominator: Instituto Brasileiro de Geografia e Estatística (IBGE)	2015
**Responsive caregiving**	Visits by national home‐visiting parenting skills programme^c^	Percentage of individuals visited by the national home‐visiting parenting skills program in the municipality in relation to the target agreed for the year	These home visits focus on strengthening parenting skills, especially for socially vulnerable parents, and help these families better understand the child development process, as well as how to behave and respond to provide adequate development. In addition, they help identify problems and difficulties and, if necessary, seek additional support services	[(Total number of individuals visited in the municipality in 2019/goal of the municipality in 2019) * 100]	Visits by national home‐visiting parenting skills programme can strengthening parenting skills, which protect the child from suboptimal early child development	Coordenação do Programa Criança Feliz/Ministry of Citizenship	2019
**Opportunities for early learning**	Coverage of daycare and preschool	Number of enrolments in daycare and preschool in relation to the total number of children under 5 years, by year and municipality of residence	Children need access to free, quality daycare and preschools, where they will receive, under adequate supervision, the stimuli and care necessary for development	[(∑ daycare enrolment and preschool enrolment)/(population under 5 years old)] * 100, per year and municipality	Coverage of daycare and preschool can protect the child from suboptimal early child development	Censo Escolar (school census)	2015
	Number of students per daycare professional	Number of students enrolled for each professional employed in public and private daycares in the municipality per year	The number of children per professional should enable all children to receive the attention, responsibility and interaction necessary for adequate development	(Number of daycare enrolments/number of daycare professionals) * 100, per year and municipality	A higher number of students per daycare professional is a risk factor for suboptimal early child development	Censo Escolar (school census)	2016
	Number of students per preschool professional	Number of students enrolled for each professional employed in public and private preschools in the municipality per year	The number of children per professional should enable all children to receive the attention, responsibility and interaction necessary for adequate development	(Number of preschool enrolments/number of preschool professionals) * 100, per year and municipality	A higher number of students per preschool professional is a risk factor for suboptimal early child development	Censo Escolar (school census)	2016
	Percentage of qualified daycare teachers	Percentage of teachers with higher education employed in public and private daycares in the municipality per year	Teachers with specific and qualified training are better prepared to understand the needs and provide early stimulation to children that supports their proper development	(Number of teachers with higher education in daycares/total number of daycare teachers) * 100, per year and municipality	A higher percentage of teachers with higher education in daycares can protect the child from suboptimal early child development	Censo Escolar (school census)	2016
	Percentage of qualified preschool teachers	Percentage of teachers with higher education employed in public and private preschools in the municipality per year	Teachers with specific and qualified training are better prepared to understand the needs and provide early stimulation to children that supports their proper development	(Number of teachers with higher education in preschools/total number of preschool teachers) * 100, per year and municipality	A higher percentage of teachers with higher education in preschools can protect the child from suboptimal early child development	Censo Escolar (school census)	2016
	Daycare educational resources	Presence of library/study room and/or playground and/or children's restroom in daycares in the municipality per year	Daycares must have adequate infrastructure for children, such as the presence of a reading room, playgrounds, adapted restrooms and educational material available. An adequate infrastructure is fundamental for the stimulation and learning of children, thus favouring complete and adequate development	(∑ number of library/study rooms, playgrounds and children's restrooms in daycare)/total daycare, by year and municipality	The availability of daycare educational resources can protect the child from suboptimal early child development	Censo Escolar (school census)	2016
	Preschools educational resources	Presence of library/study room and/or playground and/or children's restroom in schools for early childhood in the municipality per year	Preschools must have adequate infrastructure for children, such as the presence of a reading room, playgrounds, adapted restrooms and educational material available. An adequate infrastructure is fundamental for the stimulation and learning of children, thus favouring complete and adequate development	(∑ number of library/study rooms, playgrounds and children's restrooms in preschools)/total number of preschools, by year and municipality	The availability of preschool educational resources can protect the child from suboptimal early child development	Censo escolar (school census)	2016
**Security and safety**	Coverage of the national conditional cash transfer programme^b^	Percentage of families benefitting from the national conditional cash transfer programme among families in the Brazilian single registry with children under 5 years old	Cash transfer programmes protect the development of children in extreme poverty. The coverage of the national conditional cash transfer programme reflects the percentage of children in situations of poverty assisted in a social programme	(Total families benefitting from national conditional cash transfer programme with children under 5 years old in the reference month (December)/total families enrolled in the Brazilian single registry with children under 5 years old, in the reference month (December)) * 100, per municipality	The access to the national conditional cash transfer programme can protect the child from suboptimal early child development	Cadastro Único (CadÚnico/Ministry of Citizenship)	2016
	Air pollution	Estimated daily concentration of fine particulate matter (PM2.5) (ug/m3) by municipality	During pregnancy and early childhood, the rapid development of children makes them especially vulnerable to environmental stressors. Exposure to air pollution is a stressor that can affect children of all social classes, facilitating the development of childhood respiratory diseases, which can impact cognitive development and increase susceptibility to diseases in general	Estimation of the daily concentration of fine particulate matter (PM2.5) (ug/m3) from socio‐temporal models by municipality	Air pollution is a risk factor for suboptimal early child development	Brentani A et al., Child Development Center. Faculty of Medicine. University of São Paulo	2015
	Notification of violence against women	Total reported cases of any type of violence against women of childbearing age (10–49 years) in relation to the number of women in this age group in the municipality per year	Violence against women can result in several health problems such as abortion, low birth weight and prematurity. The notification of violence by the health service is a method to create a qualified service network against chronic exposure to violence, which impacts child development	(Number of cases with ICD Y09 (aggression by unspecified means) in women between 10 and 49 years old/population of women between 10 and 49 years old) * 1000, per year and municipality	The notification of violence against women create a qualified service network against chronic exposure to violence, which can protect the child from suboptimal early child development	Numerator: Sistema Nacional de Agravos de Notificação (SINAN/Ministry of Health) and denominator: Instituto Brasileiro de Geografia e Estatística (IBGE)	2015
	Notification of violence against children	Total reported cases of any type of violence against children under 5 years old in relation to the total number of children under five in the municipality per year	The notification of violence by the health service is a method to create a qualified service network against the child's chronic exposure to violence, which impacts child development	(Number of cases with ICD Y09 (aggression by unspecified means) for individuals under 5 years old/population under 5 years old) * 1000, per year and municipality	The notification of violence against children create a qualified service network against chronic exposure to violence, which can protect the child from suboptimal early child development	Numerator: Sistema Nacional de Agravos de Notificação (SINAN/Ministry of Health) and denominator: Instituto Brasileiro de Geografia e Estatística (IBGE)	2015
	Homicides	Homicide rate estimated per 100,000 inhabitants for each municipality	Early exposure to violent environments can impair childhood development, including brain development, and damage other parts of the nervous system with lifelong consequences. A safe environment with low levels of violence allows the children and their caregivers to be safe and protected, free to come and go, which permits them to explore the environment around them	(∑ number of deaths due to aggression, number of deaths caused by legal intervention, number of hidden homicides/municipal population) * 100,000, per year and municipality	A higher homicide rate is a risk factor for suboptimal early child development	Instituto de Pesquisa Econômica Aplicada (IPEA—Institute of Applied Economic Research)—Atlas da Violência (Atlas of Violence)	2017

^a^ Estratégia Amamenta e Alimenta Brasil;

^b^ Programa Bolsa Família;

^c^ Programa Criança Feliz.

## 4 DISCUSSION

As far as we know, IMAPI represents the first attempt to identify nurturing care indicators at the municipal level using routine information systems. Although other experiences measuring the environment for ECD (SEADE, 2017; UNICEF and & Countdown to 2030, 2019) or child health (Lennart Köhler & Eriksson, 2018) exist, none had used indicators at the municipal level based on the comprehensive NCF constructed into a single index as IMAPI does. IMAPI responds to the global call for developing data science initiatives to support local levels in promoting nurturing care (Richter et al., 2019; World Health Organization, World Bank Group, & United Nations Children's Fund, 2019). Our study is innovative because IMAPI not only developed a specific protocol for measuring NCF at the municipal level but also piloted it in the Brazilian context. Hence, the clear documentation of the systematic methodology followed, and lessons learned through the participatory decision‐making process can greatly assist with the dissemination of this approach into other countries.

Rooted in the evidence‐based NCF, our approach to identifying nurturing care indicators was a result of a stepwise participatory decision‐making methodology including a carefully selected group of multisectoral stakeholders and experts. This participatory process was efficient as it led to (i) the identification of indicators already used to monitor child health as well as access and quality of services in routine information systems, (ii) the inclusion of SMART properties for the stakeholders or end users of IMAPI in the calculation of the index and (iii) the consideration of the attributes of quality and limitations of each indicator.

The use of a participatory decision‐making methodology was a strength of IMAPI. Participatory decision‐making and consensus processes can effectively help stakeholders reach consensus through trust and learning within a reasonable time on a valid outcome (Devente et al., 2016). Similar participatory consensus methodologies have been used and proved effective in the selection of indicators in other areas that are highly relevant to ECD including child well‐being (McQuinn et al., 2019), breastfeeding (Pérez‐Escamilla et al., 2018) and neonatal health (Darmstadt et al., 2014). Another strength of IMAPI was the systematic engagement of key Brazilian stakeholders in the technical panels as well as national and international ECD experts to identify, select and reach consensus on indicators. Engaging with stakeholders in the decision‐making process of selecting indicators can increase the likelihood that stakeholders will support project goals and implement decisions in the long term (Devente et al., 2016).

Considering IMAPI's goal to combine the selected indicators into a single overall index and sub‐indexes for each NCF domain, taking into account an evidence‐based analytical model is considered critical to improving reliability by giving higher weight to components with stronger impact on the outcome (Köhler, 2016). This is particularly true in the context of comparing municipalities within a given country (Köhler, 2016). We articulated within the participatory decision‐making process a sound approach to account for SMART properties of each indicator considering specificity, attributes of quality and influence on ECD considering the NCF. In fact, the SMART approach has been described as a strong method to convert any type of weight assignment technique (i.e. relative/absolute) into numbers/weights (OECD, 2008; Velasquez & Hester, 2013). It also has been successfully used as a multicriteria decision analysis framework in healthcare with a focus on low‐ and middle‐income countries (Németh et al., 2019). Through the SMART process, we gained an in‐depth understanding of the different dimensions of each indicator to support the interpretation of IMAPI indexes. Hence, the SMART approach can be considered a strength of our methodology.

Furthermore, we identified some challenges related to data access and quality as well as the need to expand coverage of information and include new indicators to properly monitor the nurturing care at the municipal level. Regarding data access, even though we were using mostly routine governmental information system indicators, some of the databases were not publicly available. Thus, the request to access data to estimate multiple indicators, for example, ‘visits by national home‐visiting parenting skills programme’, ‘child immunization’ and ‘coverage of the national conditional cash transfer programme’, went through a long and bureaucratic administrative process. Furthermore, despite all the team and stakeholders' efforts in accessing data for every potential indicator, a few indicators were not made available to us, for example, ‘the number of children that are wards of the State’. For data quality, one example happened within the adequate nutrition NCF domains where indicators mainly came from the Brazilian Food and Nutrition Surveillance System (SISVAN), which collects continuous information about nutritional status and food consumption of children and adolescents receiving primary healthcare services (Mourão et al., 2019). Because of the low coverage of SISVAN across municipalities, states and regions of the country reported previously (Mourão et al., 2019) and confirmed in our study, IMAPI could only include the coverage of information on infant and young children feeding practices and nutritional status in each municipality instead of the individual indicators of feeding practices (e.g. breastfeeding, exclusive breastfeeding and quality and diversity of diet) and nutritional status(e.g. prevalence of stunting, prevalence of overweight/obesity, prevalence of low weight for age and prevalence of low weight for height) as originally planned by the research team. Due to the scarcity of information collected routinely to monitor critical factors influencing ECD outcomes in the existing national databases, two indicators from cross‐sectional research were incorporated in the adequate nutrition (‘household food insecurity’) (Gubert & Pérez‐Escamilla, 2013) and security and safety (‘air pollution’) (Brentani, 2020) domains.

Similarly, among the NCF domains, responsive caregiving was the most challenging domain to identify indicators. To date, there is no monitoring system of responsive caregiving practices in Brazil. Only one indicator for the Responsive caregiving domain was identified at the municipal level, ‘coverage of Criança Feliz home visiting programme’, that targets the most vulnerable families in Brazil (Buccini et al., 2021; Girade, 2018). Although other ECD initiatives and programmes are being implemented at the municipal level, there is no national system to collect and monitor this information for all municipalities. Thus, Criança Feliz was identified as a *proxy* of municipality's effort to supporting parenting skills and ultimately supporting responsive caregiving practices. The lack of information on responsive caregiving across countries has been reported previously (UNICEF and & Countdown to 2030, 2019). This missing information likely reflects the small financial investment to enable responsive caregiving in Brazil (Arregoces et al., 2019) and globally.

In the process of developing IMAPI, we faced some challenges that must be considered when interpreting nurturing care contexts of municipalities. First, the availability of indicators at the municipal level. Many indicators did not have disaggregated information for municipalities and were excluded from this version of IMAPI, including ‘basic sanitation’, ‘excessive alcohol consumption’ and ‘maternal mental health’. On the other hand, one advantage of using data aggregated at the municipal level is that the selection of indicators was not limited to any restrictions on Brazilian protection laws to access individual or identifiable information. Second, the lack of official data/estimation of children under 5 years old in the Brazilian population since 2016 made timeliness an important limitation for 20% of IMAPI's indicators. Fortunately, an estimation of the Brazilian population, including the population of children under 5 years old, was recently launched and will allow updates of IMAPI indicators for more recent years (Freire et al., 2019; IBGE, 2020.). Third, one challenge we can anticipate for the analytical weight when using IMAPI to follow the performance of municipalities over time is that SMART properties of indicators may also change over time (OECD, 2008). Fourth, we acknowledge that the lack of several indicators in the responsive caregiving domain is a current limitation of IMAPI.

In spite of these challenges, we were able to design and pilot a systematic methodology for identifying and selecting a set of nurturing care indicators at the municipal level. In summary, the greatest strength of IMAPI is the systematic consensus process to identify indicators using the best information available in existing Brazilian databases to monitor the domains of the NCF. The selection of IMAPI indicators was followed by the (i) use of a data engineering approach to create an index for each domain as well as a single index of the NCF and (ii) analysing whether IMAPI indexes can discriminate nurturing care environments across the 5570 municipalities in Brazil, which are described elsewhere.

## CONTRIBUTIONS

GB, MEB and MBG conceptualized the Brazilian Early Childhood Friendly Municipal Index (IMAPI) and designed the participatory decision‐making method with intellectual inputs of RPE and SIV throughout the process. JP, SC, GFC, AS, JB and JG conducted activities within the participatory decision‐making method and analysed the data. GB wrote the first draft of the manuscript, and MBG, MEB, RPE and SIV revised it critically through an iterative process. All authors approved the final version of the manuscript.

## CONFLICTS OF INTEREST

The authors declare no conflicts of interests.

## Supporting information


**Table S1.** List of institutions represented at panellists groups in the participatory decision‐making process.

**Table S2.** History of nurturing care indicators during the participatory decision‐making process of the Brazilian Early Childhood Friendly Municipal Index (IMAPI).

**Table S3.** Methodological note on the SMART criteria definition, classification and analysis.

## Data Availability

The data that support the findings of this study are available from the corresponding author upon reasonable request.
