# Supplementary material for: Nurturing care indicators for the Brazilian Early Childhood Friendly Municipal Index (IMAPI)
Source: Matern Child Nutr. 2021 May 4;18(Suppl 2):e13155. doi: 10.1111/mcn.13155 (PMC8968942; doi:10.1111/mcn.13155)
Supplement: Supplementary file 1 — Table S1. List of institutions represented at panellists groups in the participatory decision‐making process. [file MCN-18-e13155-s003.docx]

Supplementary Material 1. List of institutions represented at panelists groups in the participatory decision-making process.

| Panelists | Institutions |
| --- | --- |
| IMAPI Team | 3 Principal investigators, 6 Research assistants in Data Science & Machine Learning and Maternal- Child Nutrition and Health, and 2 Senior advisors. One senior expert is a national recognized researcher in maternal and child health-nutrition and public health in Brazil and the second expert is an international senior specialist in ECD in the USA and co-author of the ECD Lancet series that outlined the Nurturing Care Framework |
| Technical panels | General Coordination of Food and Nutrition (CGAN - Ministry of Health), General Coordination of Guarantee of Primary Care Attributes (CGAB - Ministry of Health), General Coordination of Child Health and Breastfeeding (CGSCAM - Ministry of Health), General Coordination of the National Immunization Program (CGPNI - Ministry of Health), Coordination of the School Health Program (Ministry of Health), General Coordination of Food and Nutrition Education (CGEAN - Ministry of Citizenship), Secretariat for Evaluation and Information Management (SAGI - Ministry of Citizenship), National Secretariat for the Promotion of Human Development (Ministry of Citizenship), National Institute of Studies and Research (INEP), Yale School of Public Health, Federal District Planning Company (CODEPLAN), Federal District Health Secrariat, Brasiliense Happy Child Program, Parliamentary Front of Early Childhood, United Nations Children's Fund (UNICEF), Pan American Health Organization (PAHO), Recife Health Secrariat, Institute of Health of São Paulo, Todos pela Educação NGO. |
| Expert panels | Yale School of Public Health, Harvard School of Public Health, Harvard University Center on the Developing Child, China Development Research Foundation, Institute of Health of São Paulo, Federal University of Ceará, Maria Cecilia Souto Vidigal Foundation, University of São Paulo, Federal University of Minas Gerais, National Secretariat for the Promotion of Human Development (Ministry of Citizenship), Teaching and Research Institute (Insper), Institute of Integral Medicine Professor Fernando Figueira, Science for Early Childhood Nucleus (NCPI). |
