# Supplementary material for: Nurturing care indicators for the Brazilian Early Childhood Friendly Municipal Index (IMAPI)
Source: Matern Child Nutr. 2021 May 4;18(Suppl 2):e13155. doi: 10.1111/mcn.13155 (PMC8968942; doi:10.1111/mcn.13155)
Supplement: Supplementary file 2 — Table S2. History of nurturing care indicators during the participatory decision‐making process of the Brazilian Early Childhood Friendly Municipal Index (IMAPI). [file MCN-18-e13155-s001.docx]

Supplementary Material 2. History of nurturing care indicators during the participatory decision-making process of the Brazilian Early Childhood Friendly Municipal Index (IMAPI).

| **Nome of the indicators** | **Summary of modifications** | | | | |
| --- | --- | --- | --- | --- | --- |
|  | **ECD Indicators Version 1** | **ECD Indicators Version 2** | **ECD Indicators Version 3** | **ECD Indicators Version 4** | **ECD Indicators Version 5** |
| Prenatal care consultations |  | M | R | R | R |
| Early start of prenatal care |  | A | R | R | I |
| Congenital syphilis |  | A | R | R | I |
| C-Section |  | A | R | I | I |
| Home visits in the first 10 days of child’s life |  | R | M | I | I |
| Child hospitalization for pneumonia or gastroenteritis |  | R | R | R | I |
| Adolescent pregnancy |  | R | I | I | I |
| Low birth weight |  | I | R | I | I |
| Child mortality |  | I | R | I | I |
| Preventable deaths in children under 1 years old |  | A | R | I | M |
| Prematurity |  | D | R | I | I |
| Maternal mortality |  | D | R | I | R |
| Coverage of child immunization |  | I | R | M | I |
| Coverage of Primary Health Care |  |  | A | I | I |
| Coverage of information on child feeding practices ^a^ |  | I | M | I | R |
| Severe Household Food Insecurity |  | I | R | I | I |
| Brazilian Breastfeeding and Feeding Strategy (*Estratégia Amamenta e Alimenta Brasil)* |  | A | R | I | I |
| Coverage of information on child nutritional status ^b^ |  | I | M | D | R |
| Coverage of daycare and preschool^c^ |  | M | R | R | I |
| Number of students per daycare professional |  | A | R^d^ | M | M |
| Number of students per preschool professional |  |  | A^d^ | M | M |
| Percentage of qualified daycare teachers |  | A | R^e^ | M | I |
| Percentage of qualified preschool teachers |  |  | A^e^ | M | I |
| Daycare educational resources |  |  | A^f^ | M | I |
| Preschool educational resources |  |  | A^f^ | M | I |
| Visits by national home-visiting parenting skills program (*Criança Feliz Program)* |  | D & R | R | I | M |
| Notification of violence against children^h^ |  | D & R | R | R | R |
| National conditional cash transfer program *(Bolsa Família Program)* |  | D | R | I | I |
| Air pollution |  | I | R | D | I |
| Notification of violence against women^h^ |  |  |  | A | R |
| Homicides^g^ |  |  |  |  | A |
| Additional indicators |  |  |  |  |  |
| Total municipality population |  | I | R | I | I |
| Size of the municipality |  |  | A | I | I |
| Total population of children under 5 years old |  | R | R | I | R |
| Proportion of children under 5 in relation to the total population |  | I | I | I | R |
| Births per year |  | I | R | I | I |
| Region of Brazil |  | A | I | I | I |
| Federative Unit |  |  | A | I | I |
| Children under 5 years old in vulnerability |  | I | I | R | I |
| Proportion of children under 5 years old in vulnerability in relation to the total number of children |  |  |  | A | I |
| Human Milk Bank |  | R | I | I | I |
| Child-friendly hospital |  | M | I | I | I |
| Water system supply |  | D & R | R | D | I |
| Sewage system |  | D & R | R | D | I |
| Excluded | | | | | |
| Treatment for HIV + pregnant women |  | E |  |  |  |
| Consumption of Iodized salt to prevent iodine deficiency |  | E |  |  |  |
| Family planning |  | A | E |  |  |
| Prevalence of anemia in young children |  | I | E |  |  |
| Child mortality rate |  | I | E |  |  |
| Number of child growth and development monitoring consults in the first 6 months child’s life |  | R | R | E |  |
| Support for parents through groups and home visits |  | E |  |  |  |
| Early start of breastfeeding |  | I | E |  |  |
| Children play with objects at home |  | E |  |  |  |
| Childcare organization |  | E |  |  |  |
| Reasons why parents left children under 4 years old under the responsibility of others |  | E |  |  |  |
| Affordable and good quality daycare |  | E |  |  |  |
| Other programs, in addition to visits by national home-visiting parenting skills program ^c^, that aim to improve Early Child Development |  | A | E |  |  |
| Maternal mental health |  | R | I | E |  |
| Municipal information and communication on  ECD and services |  | M | R | I | E |
| Inadequate child supervision |  | D | R | I | E |
| Stimulation for early learning |  | D | R | I | E |
| Children's books at home |  | D | I | I | E |
| Positive discipline |  | E |  |  |  |
| Birth registration |  | I | E |  |  |
| Number of “corporate citizenship” companies (*Empresas cidadãs*) |  | D | E |  |  |
| Compliance with the conditionalities of Conditional Cash Transfer Program |  | A | E |  |  |
| Maternity leave |  | D & R | I | E |  |
| Abusive alcohol consumption by adults |  | D | R | I | E |
| Household expenses with Early Child Development (daycares) |  | E |  |  |  |
| Children with disabilities |  | E |  |  |  |
| Cost of growth deficit |  | E |  |  |  |
| Prevalence of orphans |  | E |  |  |  |
| Policies covering Early Child Development |  | E |  |  |  |
| Delay in Early Child Development |  | E |  |  |  |
| Disparities by gender and residence |  | E |  |  |  |
| Composite measure of the risk of poor development due to exposure to growth deficit and extreme poverty |  | E |  |  |  |
| Government expenditure with Early Child Development |  | I | E |  |  |
| Municipal GDP |  | M | E |  |  |
| Homes headed by women |  | M | E |  |  |
| Municipal Deprivation Index |  | A | E |  |  |
| Unemployment rate |  | I | R | E |  |
| Municipal Human Development Index |  | I | I | I | E |
| Fertility rate |  | I | I | I | E |
| Low maternal educational level |  | I | I | I | E |
| Municipal adherence to social programs such as support for people with disabilities (‘Benefício de Prestação Continuada’), Primary Care packages (‘Rede Cegonha’) |  | A | I | M | E |
| Legal Amazônia area |  | A | M | I | E |
| Indigenous area |  |  | A | I | E |
| Semi-arid area |  | A | M | I | E |
| Total of indicators | Indicators = 35; Additional indicators = 32 | Indicators = 48; Additional indicators = 20 | Indicators = 35; Additional indicators = 19 | Indicators = 35; Additional indicators = 20 | Indicators = 31; Additional indicators = 13 |

**Color-code**: Good Health domain/yellow, Adequate Nutrition domain/pink, Responsive Care domain/green, Opportunities for Early Learning domain/red, Safety and Security domain/blue; Additional domain/purple; Excluded/grey.

**Summary of modifications**: indicator remained unchanged (I); indicator was moved to another domain of the NCF (D); indicator was modified (i.e., broken down into different indicators, the calculation method changed, the data source changed) (M); indicator was excluded (E); new indicator was added (A); indicator was renamed to facilitate understanding (R)

***Italic:*** Portuguese name of the programs

**Methodological notes:**

^a^ represented by 3 indicators: breastfeeding, exclusive breastfeeding and quality and diversity of diet

^b^ represented by 4 indicators: prevalence of stunting, prevalence of overweight/obesity, prevalence of low weight for age, prevalence of low weight for height.

^c^ represented by 3 indicators: Daycare coverage rate, Coverage of daycare in relation to the population of 0 to 3 years and coverage of preschool in relation to the population of 4 to 5 years

^d^ represented by one indicator: Number of students per professional in daycares and preschools

^e^ represented by one indicator: Percentage of qualified teachers in daycares and preschools

^f^ represented by one indicator: Daycare and preschool educational resources

^g^ included ‘Homicide’ as a proxy of an unsafe community environment for ECD

^h^ interpretation of ‘notification of violence against children and women’. There was a discussion whether these indicators should be interpreted as protective (the more notification, the more relevant the issue is for that community, thus more supportive services would be in place) or risk (the more notification, the more violence occurs in that community) for ECD. This is because, although in Brazil the notification of violence against children and women is mandatory, a notified case or suspect case is not a formal complaint, instead is an instrument for guarantee rights; thus, a protective community factor. To confirm this hypothesis, we selected ‘Homicides’ an indicator within the same NCF domain, serving as a proxy of an unsafe community. The negative correlation between ‘Homicides’ and ‘Notification of violence against women’ (r=-0.11 p<0.001) and ‘Notification of violence against children’ (r=-0.04 p<0.05) further confirmed the hypothesis of the notification as protective community factor for ECD. Thus, the term ‘notification’ was included in both indicators of violence.
