# Supplementary material for: Nurturing care indicators for the Brazilian Early Childhood Friendly Municipal Index (IMAPI)
Source: Matern Child Nutr. 2021 May 4;18(Suppl 2):e13155. doi: 10.1111/mcn.13155 (PMC8968942; doi:10.1111/mcn.13155)
Supplement: Supplementary file 3 — Table S3. Methodological note on the SMART criteria definition, classification and analysis. [file MCN-18-e13155-s002.docx]

| Supplementary material 3. Methodological note on the SMART criteria definition, classification and analysis. |
| --- |
| 1. **Specific (S):** confirm that the indicator belongs to the Nurturing Care Framework (NCF) domain |
| **Question:** “Does **[name of the indicator]** belong to the domain to which it was initially allocated?”  **Example:** “Does low birth weight indicator belong to “Good Health” domain? |
| **Expert responses**: Yes/No. If no, the expert was asked to indicate the NCF domain and justify the suggested change.  **Analysis:** percentage of agreement among the expert panelist |
| **2. Measurable (M):** measure the quality of indicator bases on pre-defined attributes*: periodicity of data^a^, data source^b^, access to data^c^, population profile^d^. This criterion was assessed from two perspectives: Expert Panel and IMAPI Team. |
| 2.1. Expert Panel: |
| **Question:** “How important is **[name of the attribute]** to the overall quality of the **[name of the indicator]**?”  **Example:** “How important is periodicity to the overall quality of low birth weight? |
| **Responses**: 5-point Likert scale  5 = very important  4 = important  3 = neutral  2 = not very important  1 = not important  **Authors’ methodological note:** This question was asked for each attribute (periodicity of data, data source, access to data, population profile). |
| 2.2 IMAPI Team: |
| **Question**: “Score **[name of the indicators]** according to the four attributes of quality evaluated by the experts”.  **Example:** “Score low birth weight indicator according to the four attributes of quality evaluated by the experts” |
| **Responses**:  a) periodicity of data 1 = onetime, 3 = biannual, 5 = monthly, semiannual, or annual (W1 Periodicity);  b) data source 1 = projection, 3 = research, 5 = system (W1 Source);  c) data access 1 = private, 3 = upon request to the government, 5 = available for download via internet (W1 Access);  d) population profile 1 = specific population groups, 3 = population using the Brazilian Unified Health System (SUS) or the education system, 5 = Entire Brazilian population (W1 Population profile)  **Analysis:** Criterion M for each indicator consisted of the mean between the average of M1 and W1 of each quality attribute. The formula for calculating the criterion M = {[(M1 Periodicity + W1 Periodicity)/2] + [(M1 Source + W1 Source)/2] + [(M1 Access + W1 Access)/2] + [(M1 Population profile + W1 Population profile)/2]}/4. |

| **3. Achievable (A)**: assess the level of governance of the municipal sphere in modifying the indicators for greater impact on Early Child Development (ECD) |
| --- |
| **Question:** “What is the governability of the municipality to modify **[name of the indicator]** to enhance the impact on ECD?”  **Example:** “What is the governability of the municipality to modify **low birth weight indicator** to enhance the impact on ECD? |
| **Expert responses**: 5-point Likert scale  5 = Complete governability: the actions necessary to modify this indicator depend completely on the municipal level;  4 = Partial governability: the actions necessary to modify this indicator depend mostly on the municipal level;  3 = Average governability: the actions necessary to modify this indicator depend in part on the municipal sphere, and part on the state or federal sphere;  2 = Almost no governability: the actions necessary to modify this indicator depend mostly on the state or federal sphere;  1 = No governability: the actions necessary to modify this indicator depend completely on the state or federal level |
| **Analysis:** Criteria A for each indicator consisted of the average of expert’s responses on the Likert scale |
| **4.** **Relevant (R)**: assess how much changes in indicators could impact ECD outcome |
| **Question**: “If **[name of the indicator]** performed better, how much it would improve ECD outcomes?”  **Example:** “If **low birth weight indicator** performed better, how much it would improve ECD outcomes?” |
| **Expert response**: 5-points Likert scale  5 = Highly determinant – positive changes in this indicator would greatly improve ECD;  4 = Very determinant – positive changes in this indicator would improve ECD;  3 = Moderately determinant – positive changes in this indicator would somehow improve ECD;  2 = Minimally determinant – positive changes in this indicator would barely improve ECD;  1 = Almost not determinant – positive changes in this indicator would not improve ECD at all.  **Analysis:** Criteria R for each indicator consisted of the average of experts’ responses on the Likert scale |
| **5**. **Time-bound (T)**: assess the time necessary for changes in an indicator to improve ECD outcome. |
| **Question**: “If **[name of the indicator]** performed better, how long you estimate it would take to improve the ECD outcomes?”  **Example:** **“**If **low birth weight indicator** performed better, how long you estimate it would take to improve ECD outcomes?”  **Expert responses**: Likert scale 3 points  5 = short term (up to 1 year)  3 = medium term (2 to 3 years)  1 = long term (4 or more years)  **Analysis:** Criteria T for each indicator consisted of the average of experts’ responses on the Likert scale |
| *Operational definitions for attributes of quality: ^a^periodicity of data: period in which the data is published. Data periodicity can influence the index update, ^b^data source: the data comes from systems, research or prediction/projections. Data source can influence to what extent the index represents the real situation; ^c^access to data: data can be accessed in public or private way. The way of accessing the data can influence the index sustainability; ^d^ population profile: the population profile can influence the representativeness of the index in relation to the total population of the municipality. |
